# Resourcefulnsss of Texas Doctors

**Published:** 1906-09

**Authors:** C. H. Hughes

**Affiliations:** St. Louis


For Texas Medical Journal.

### Resourcefulnsss of Texas Doctors.

LETTER FROM DR. HUGHES.

St. Louis, August 11, 1906.

Dear Doctor Daniel: I was in your State last year as far as Dallas, and I regret I did not have time to visit you. I like the Texas doctors,—her handsome women, her noble, virile men, and her handsome houses and her new energy. The Texas country doctors are resourceful and fertile in therapeutics and surgical experiments, especially the former generations, when the drug and instrument stores were fewer and farther between than they are now. They could mash potato-bugs with a common flour or dough roller, and mix with lard as a substitute for *emplastrum cantharides,* and extemporize handy catheters for grave emergencies out of green wheat-straws, greased in the stem and moistened at the insertion-end, and make use of many other valuable substitute resources in pressing emergencies of practice which I will not take time now to enumerate.

Country doctors of other Southwestern States are likewise resourceful. I remember one instance where at tardy labor with the fetal head somewhat impacted in the inferior strait, the doctor in attendance improvised a forceps-blade from a hickory corsetboard, such as our mothers and some of the grandmothers of some of the present-day doctors used to wear in their stays, shaving it thinner with his jack-knife, and using it as a vectis. It was from a Texas doctor that I got some of the first suggestions of my early therapeutics in country practice when I began as a general practitioner. The doctor's name was Beall,* from Fort Worth. Is he still living there or anywhere in Texas? It was a Texas or Missouri student who wrote a good thesis in 1859 on *Phytolacca Decandra* in the management of rheumatism or obesity, and we have *phytolin* from that thesis.

Very truly yours,

C. H. HUGHES.

*Prof. Elias J. Beall, M. D., Emeritus Professor Practice of Medicine, University of Fort Worth. Editor.

